# Supplementary material for: Cardiac dopamine D1 receptor triggers ventricular arrhythmia in chronic heart failure
Source: Nat Commun. 2020 Aug 31;11:4364. doi: 10.1038/s41467-020-18128-x (PMC7459304; doi:10.1038/s41467-020-18128-x)
Supplement: Supplementary file 1 — Supplementary Information [file 41467_2020_18128_MOESM1_ESM.pdf]

Supplementary Information

Cardiac Dopamine D1 Receptor Triggers Ventricular Arrhythmia in Chronic Heart Failure

Yamaguchi T., *et al.*

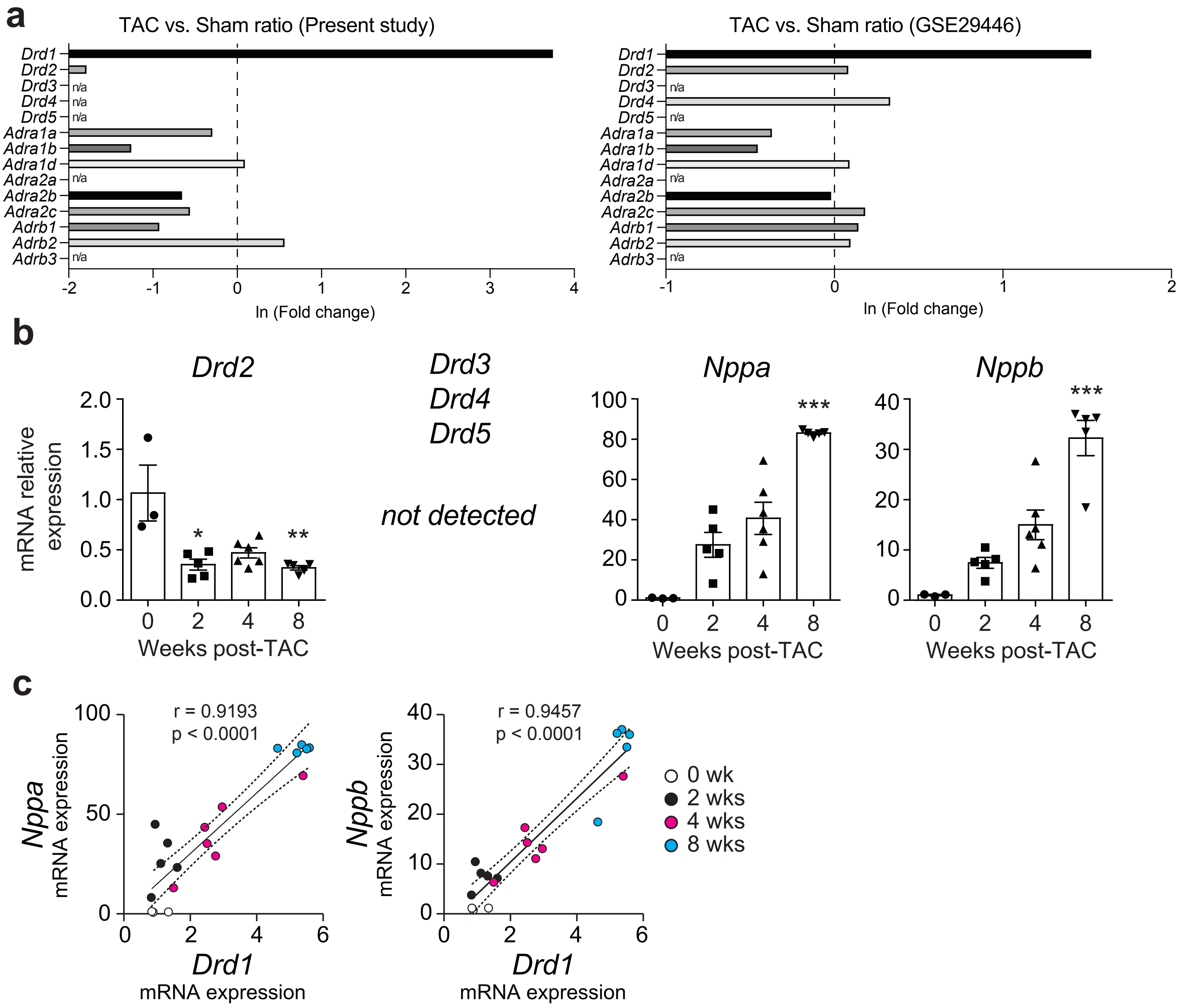

Supplementary Figure 1: Gene expression of catecholamine receptors in failing heart. (**a**) Bar graph showing the natural logarithm transformed fold ratio (ln) of catecholamine receptors expression levels between TAC-operated and sham-operated hearts observed in RNA-seq data. (Left) Our present study. (TAC n=1, Sham n=2) (Right) Publicly available RNA-seq data from GSE29446 (TAC n=1, Sham n=2). n/a, not applicable. (**b**) Bar graph showing relative expression of *Drd2-5*, *Nppa* and *Nppb* gene in the heart tissue before and after the TAC operation analyzed by qPCR (0 week: Sham-operated, n=3; 2 weeks, n=5; 4 weeks, n=6; 8 weeks, n=5). Data is shown as mean and s.e.m. Statistical significance was determined by Kruskal-Wallis test followed by Dunn's multiple comparisons test. *Drd2* *p = 0.030, **p = 0.008; *Nppa* ***p = 0.0008; *Nppb* ***p = 0.0009 versus 0 week. (**c**) Correlation plots between the expression of *Drd1* and *Nppa* (left) and *Nppb* (right) gene. Linear positive correlation and 95% confidence intervals are shown, and the person's r and p values are indicated. Each dot color represents the different time points after TAC operation.

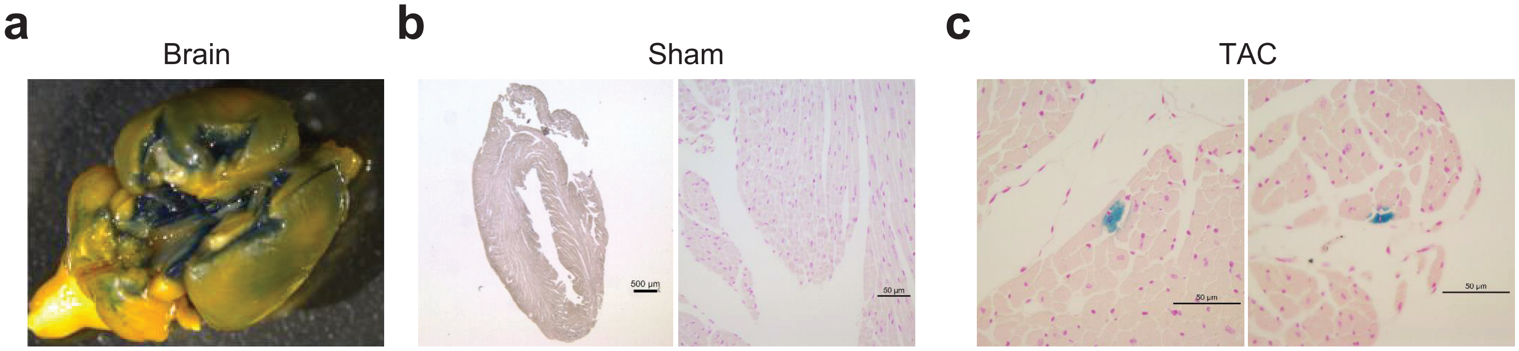

Supplementary Figure 2: LacZ stainings of heart and brain tissue. (**a**) Representative image of the LacZ staining (blue) of the whole brain from *D1R-tTA/TRE-D1R/lacZ* mice as a positive control. (**b**) Representative images of the LacZ staining of heart tissue section from sham-operated *D1R-tTA/TRE-D1R/lacZ* mice. Nucleus is counterstained in magenta. Scale bar 500 µm (left), 50 µm (right). (**c**) Representative images of LacZ-positive (D1R-positive) CMs of the heart tissue from 4 weeks after TAC-operated *D1R-tTA/TRE-D1R/lacZ* mice. Nucleus is counterstained in magenta and LacZ-positive cells in blue. Scale bar 50 µm (left). We have repeated the experiments twice with similar results to confirm the reproducibility of these representative images in (b) and (c).

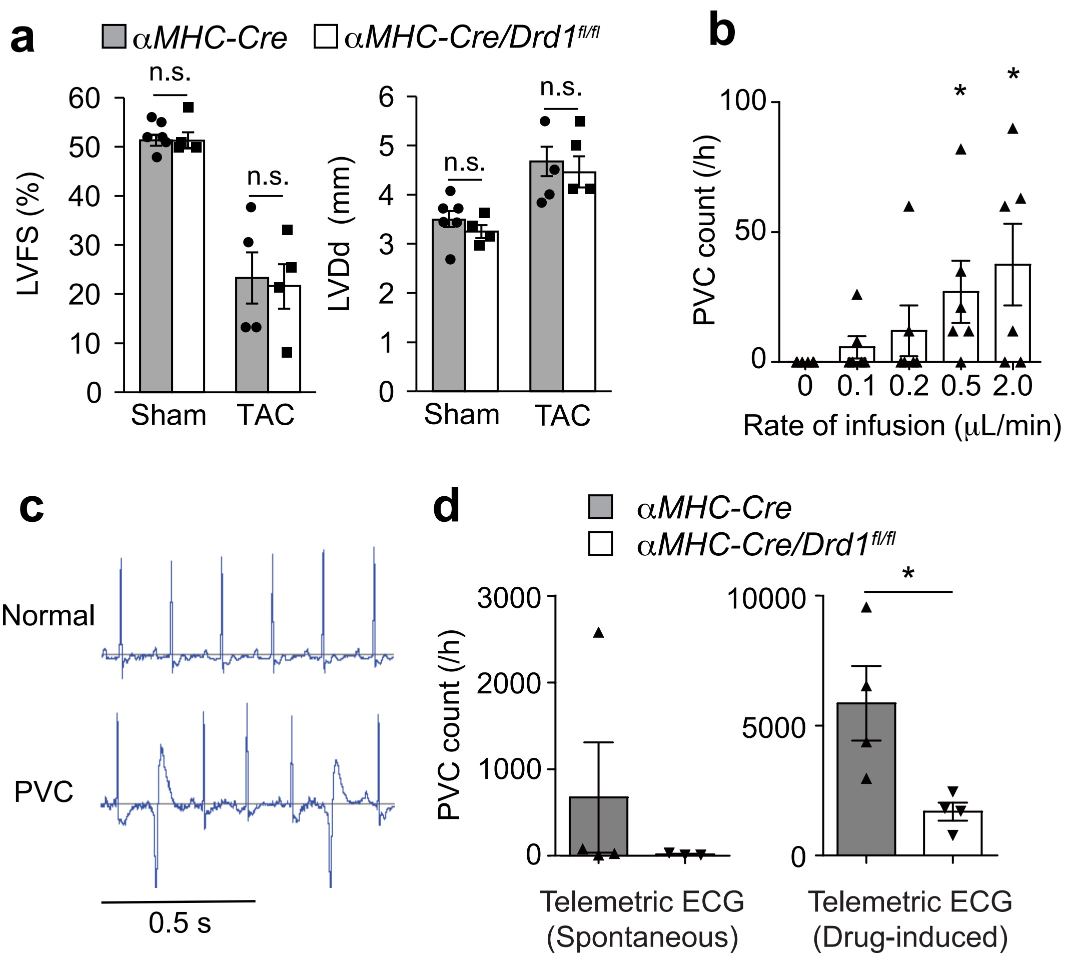

Supplementary Figure 3: Functional analyses of cardiac specific D1R deleted mice. (**a**) Cardiac function of sham- or TAC-operated *αMHC-Cre* or *αMHC-Cre/Drd1fl/fl* mice assessed by echocardiogram 8 weeks after the operation (sham-*α MHC-Cre*, n=6; sham-*αMHC-Cre/Drd1fl/fl*, n=4; TAC-operated *αMHC-Cre*, n=4; TAC-operated *α MHC-Cre/Drd1fl/fl*, n=4). LVFS, LV fractional shortening; LVDd, LV end-diastolic dimension. Data is shown as mean and s.e.m. Statistical analysis was performed by Mann-Whitney U test between two groups. n.s., not significant. (**b**) Bar graph showing the frequency of PVC in TAC-operated *αMHC-Cre* mice recorded by surface electrocardiogram (ECG) (n=6) while a mixture of dopamine and caffeine was injected intravenously at the indicated rate. Data is shown as mean and s.e.m. Statistical significance was determined by Kruskal-Wallis test followed by Dunn's multiple comparisons test. 0.5 μL/min *p = 0.032, 2.0 μL/min *p = 0.049 versus 0 μL/min group. (**c**) Representative telemetric ECG recordings of the normal sinus rhythm (Normal) and PVC in awake mice. (**d**) Bar graph showing the frequency of spontaneous and drug-induced PVC recorded by telemetric ECG in awake *αMHC-Cre* and *αMHC-Cre/Drd1fl/fl* mice after the TAC operation. (*αMHC-Cre* (TAC), n=4; *αMHCCre/ Drd1fl/fl* (TAC), n=4) Data is shown as mean and s.e.m. Statistical significance was determined by Mann-Whitney U test. *p = 0.029 between two groups.

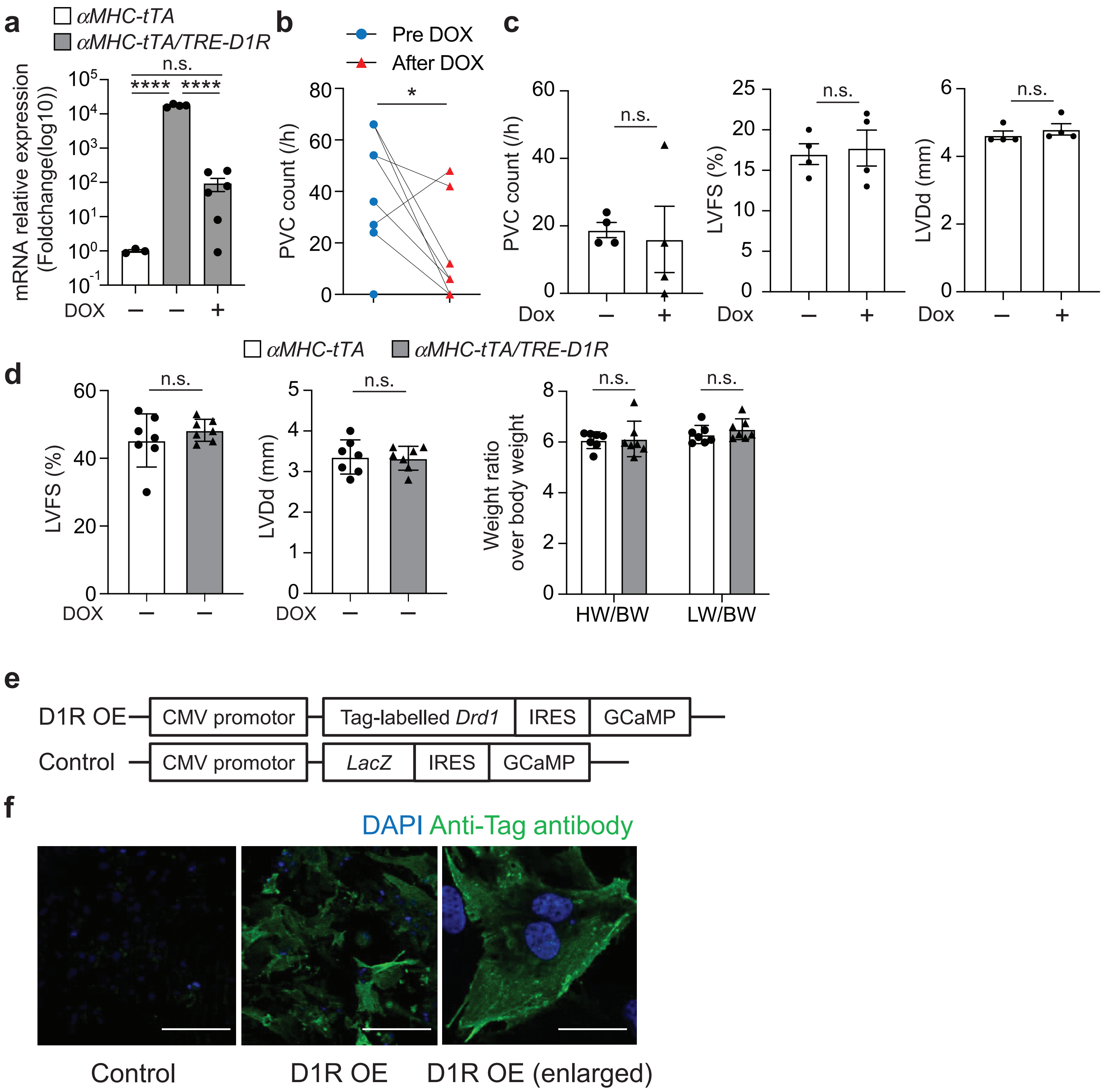

Supplementary Figure 4: Characteristics of cardiac D1R overexpression in vivo and in vitro. (**a**) Bar graph showing relative expression of *Drd1* in heart tissue from *αMHC-tTA* (control) and *αMHC-tTA/TRE-D1R* mice with and without DOX administration analyzed by qPCR. (control, n=3; *αMHC-tTA/TRE-D1R* without DOX administration, n=4; *αMHC-tTA/TRE-D1R* with DOX administration, n=6) Data is shown as mean. Statistical significance was determined by One-way ANOVA test followed by Tukey's multiple comparisons test. ****p<0.0001 between two groups. n.s., not significant. (**b**) Dot plots showing the frequency of drug-induced PVC in *αMHC-tTA/TRE-D1R* mice under anesthesia before and after DOX administration (n=8). The dots evaluated from same mice are connected by bars each other. Statistical significance was determined by Wilcoxon signed-rank test. *p = 0.047 between two groups. (**c**) Bar graph showing the frequency of drug-induced PVC in TAC-operated wild-type mice (8 weeks after the TAC surgery) under anesthesia with or without 2 weeks DOX administration (n=4, each). Data is shown as mean and s.e.m. Statistical analysis was performed by Mann-Whitney U test between two groups. n.s., not significant. (**d**) Bar graph showing cardiac function assessed by echocardiogram (left and middle), heart and lung weight ratio (right) of *αMHC-tTA/TRE-D1R* mice without DOX administration (n=7 each). LVFS, LV fractional shortening; LVDd, LV end-diastolic dimension; HW, Heart weight; LW, Lung weight; BW, Body weight. Data is shown as mean and s.e.m. Statistical analysis was performed by Mann-Whitney U test between two groups. n.s., not significant. (**e**) Construction of adenoviral vector system for overexpression of D1R and LacZ as a control. D1R OE, D1R Overexpression. (**f**) Immunocytochemistry of D1R-overexpressing CMs using anti-Tag antibody. Scale bars, 100 μm and 20 μm (enlarged figure) respectively. We have performed this experiment to confirm the success of adenoviral infection at once.

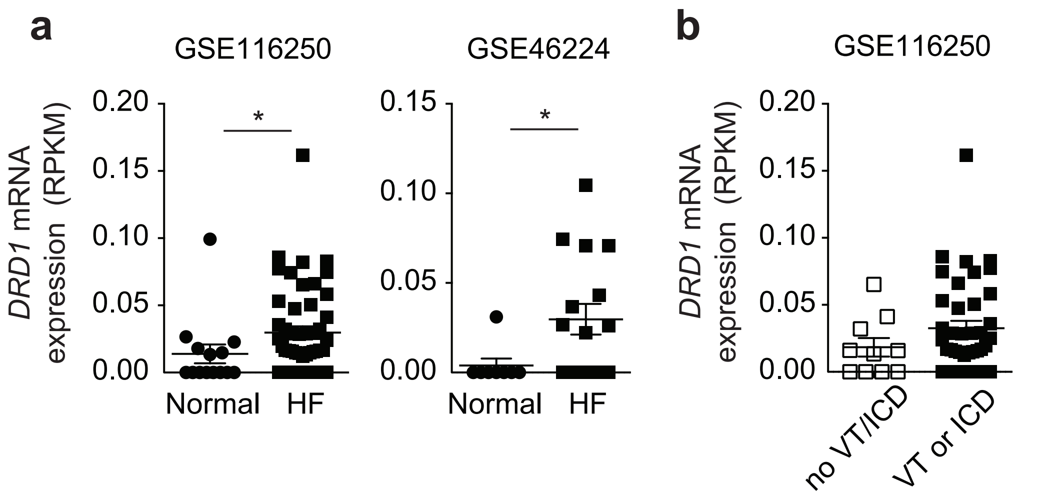

Supplementary Figure 5: The expression of *DRD1* gene in heart tissues of chronic heart failure. (**a**) Dot plots showing the expression of *DRD1* gene in whole human heart tissue evaluated by RNA-seq dataset (GSE116250 and GSE46224). Data from each patient are plotted individually (patients with normal cardiac function (Normal), patients with cardiomyopathy (HF)) (GSE116250: Normal n=14; HF n=50, GSE46224: Normal n=8; HF n=16). The mean and s.e.m. of gene expression levels are indicated by bars. Statistical significance was determined by Mann-Whitney U test. GSE116250 *p = 0.032, GSE46224 *p = 0.047 between two groups respectively. (**b**) Dot plots showing the expression of *DRD1* gene in the heart tissue from patients with cardiomyopathy evaluated by RNA-seq (GSE116250). Data from each patient are plotted individually (patients without history of ventricular tachycardia and an implantable cardioverter defibrillator device (no VT/ICD) (n=10), patients with history of ventricular tachycardia and/or with an implantable cardioverter defibrillator device (VT or ICD) (n=40)). The mean and s.e.m. of gene expression levels are indicated by bars.

Figure. 2b

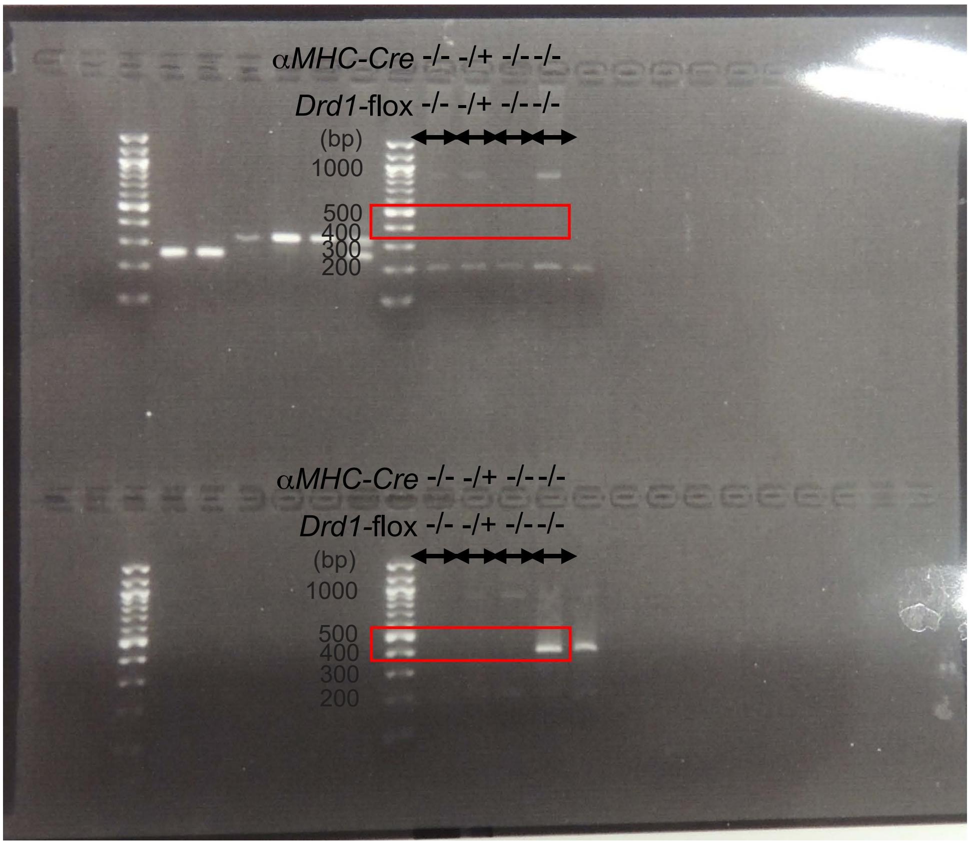

Figure. 3f p-RyR S2808

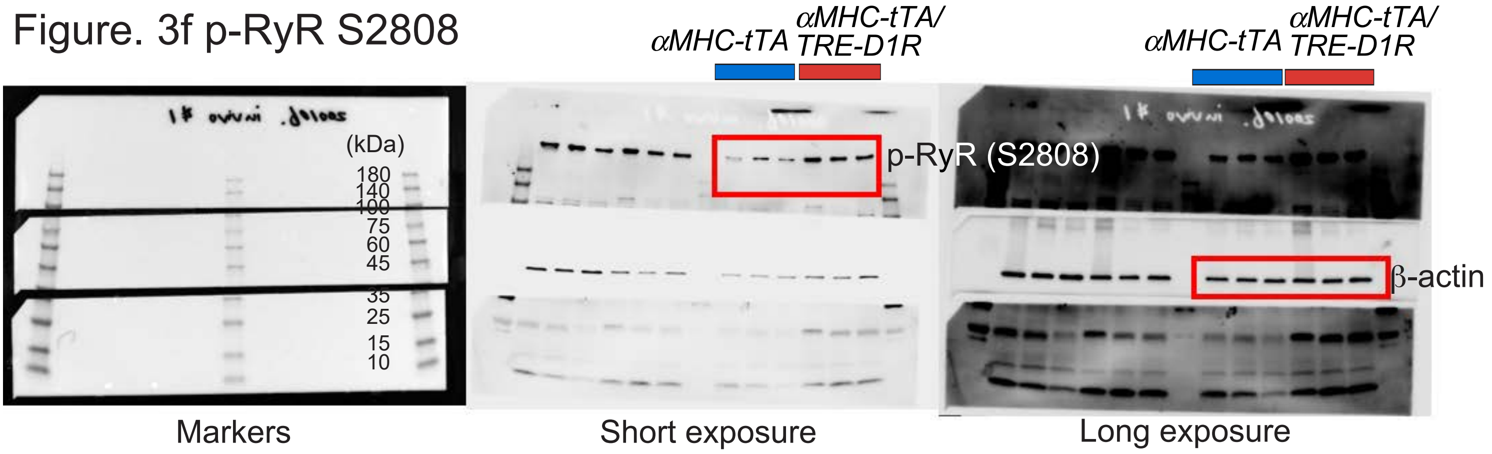

Figure. 3f p-RyR S2814

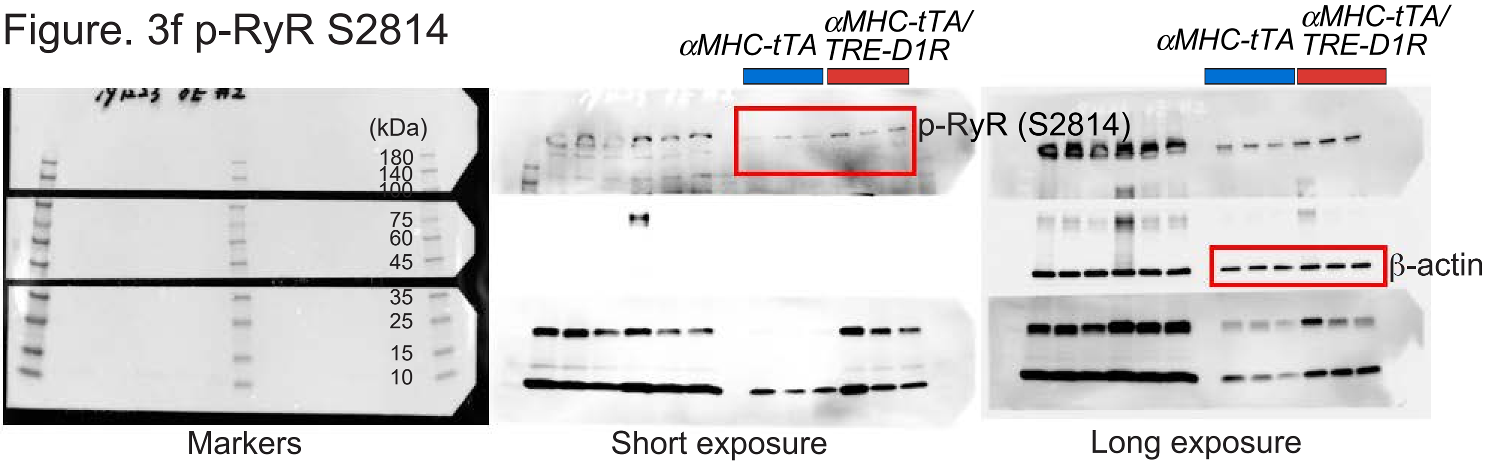

Supplementary Figure 6: Unprocessed scans of gel and immunoblots. Full scans of gel and immunoblots in Fig. 2b and Fig. 3f. Red squares indicate the scan areas shown in Fig. 2b and 3f.

Supplementary Table1. The data of sham- or TAC-operated heart

Weeks post-TAC	BW	HW	LW	HW/BW	LW/BW	LVDd	LVFS
0 week	27.1±0.7	134±8	143±1	5.0±0.2	5.3±0.2	3.0±0.1	54±2
2 weeks	25.5±1.0	154±9	142±5	6.1±0.5	5.6±0.4	3.2±0.1	38±2
4 weeks	26.1±0.6	210±11	205±31	8.1±0.3	7.8±1.1	3.0±0.3	35±10
8 weeks	24.9±1.3	278±3*	475±18	11.3±0.5**	19.1±0.5*	4.4±0.1	13±1*

Supplementary Table 1: The data of sham- or TAC-operated heart.

Physical and echocardiographic parameters of TAC-operated mouse. Body weight, heart weight, and lung weight were measured 2, 4, 8 weeks after the TAC operation or pre-operation (shown as 0 week). Cardiac function of sham- or TAC-operated wild type mice were also assessed by echocardiogram 2, 4, 8 weeks after the operation or 8 weeks after the sham operation (shown as 0 week) (n=3, each). Data is shown as mean and s.e.m. Statistical significance was determined by Kruskal-Wallis test followed by Dunn's multiple comparisons test. HW/BW; **p=0.009, HW; *p=0.01, LW/BW; *p=0.04, LVFS; *p=0.02 versus 0 week.

Supplementary Table 2. The data of heart of *αMHC-Cre* and *αMHC-Cre/Drd1fl/fl* mice

	BW	HW	LW	HW/BW	LW/BW
αMHC-Cre	23.6±0.7	127±2	132±7	5.4±0.2	5.6±0.4
αMHC-Cre/Drd1fl/fl	22.1±0.3	112±3	127±4	5.1±0.1	5.8±0.2

Supplementary Table 2: The data of heart of *αMHC-Cre* and *αMHC-Cre/Drd1fl/fl* mice.
Physical and echocardiographic parameters of *αMHC-Cre* and *αMHC-Cre/Drd1fl/fl* mice. Body weight, heart weight, and lung weight of 8 weeks old *αMHC-Cre* and *αMHC-Cre/Drd1fl/fl* mice were measured (n=5, each). Data is shown as mean and s.e.m. Statistical analysis was performed by Mann-Whitney U test between two groups and there was no significant difference.

Supplementary Table 3. The data of heart of *αMHC-tTA* and *αMHC-tTA/TRE-D1R* mice

	BW	HW	LW	HW/BW	LW/BW	LVDd	LVFS	IVS	PW
αMHC-tTA	21.8±0.8	133±6	137±4	6.1±0.1	6.3±0.1	3.4±0.1	45±3	0.8±0.0	0.8±0.0
αMHC-tTA/TRE-D1R	21.8±0.5	134±9	142±6	6.1±0.2	6.5±0.1	3.3±0.1	49±1	0.8±0.1	0.8±0.0

Supplementary Table 3: The data of heart of *αMHC-tTA* and *αMHC-tTA/TRE-D1R* mice.

Physical and echocardiographic parameters of *αMHC-tTA* and *αMHC-tTA/TRE-D1R* mice. Body weight, heart weight, and lung weight were measured and cardiac function were also assessed by echocardiogram in age of 8 weeks (n=7, each). Data is shown as mean and s.e.m. Statistical analysis was performed by Mann-Whitney U test between two groups and there was no significant difference.

BW, Body weight; HW, Heart weight; LW, Lung weight; LVDd, Left ventricular end-diastolic diameter; LVFS, Left ventricular fractional shortening; IVS, Interventricular septum; PW, Posterior left ventricular wall thickness

Supplementary Table 4. Patient demographics and characteristics.

Diagnosis	Occasion	LVDd	LVDs	LVEF	ICD/CRTD	VT	VF	CRTD	ICD	Antiarrhythmic drugs
DCM	LVAD	77	75	9	no	Yes	no	no	no	Amiodarone 200mg, Bisoprolol 1.25mg
DCM	LVAD	79	78	9	Yes	Yes	no	Yes	no	Amiodarone 100mg
DCM	LVAD	89	83	8	no	Yes	no	no	no	Amiodarone 200mg, Carvedilol 1.25mg
Sarcoidosis	LVAD	81	71	7	Yes	Yes	no	Yes	no	Carvedilol 7.5mg, Sotarol 160mg
DCM	LVAD	59	57	19	Yes	no	no	Yes	no	Amiodarone 200mg, Carvedilol 5mg
Carditis	LVAD	73	67	23	Yes	Yes	no	no	Yes	Amiodarone 200mg, Carvedilol 40mg
DCM	LVAD	73	68	10	no	no	no	no	no	Amiodarone 100mg, Bisoprolol 1.25mg
DCM	LVAD	76	70	17	no	no	no	no	no	Amiodarone 100mg, Carvedilol 2.5mg
DCM	LVAD	68	64	20	no	no	no	no	no	Carvedilol 40mg
DCM	Transplantation	64	60	14	no	no	no	no	no	Carvedilol 2.5mg
DCM	LVAD	76	72	13	no	no	no	no	no	Carvedilol 2.5mg
AML	Autopsy	41	26	67	no	no	no	no	no	none

Supplementary Table 4: Patient demographics and characteristics

At sampling, diagnosis, occasion of sample collection, cardiac function assessed just before sample collection, history of electronic device implantation, history of arrhythmias, and information of antiarrhythmic drugs prescribed just before sample collection were shown. DCM, Dilated cardiomyopathy; AML, Acute myeloid leukemia; LVAD, Left ventricular assist device; LVDd, Left ventricular end-diastolic diameter; LVDs, Left ventricular systolic diameter; LVEF, Left ventricular ejection fraction; ICD, Implantable cardioverter defibrillator; CRTD, Cardiac resynchronization therapy defibrillator; VT, Sustained ventricular tachycardia; VF, Ventricular fibrillation; PM, Pacemaker; AVB, Atrioventricular block

Supplementary Table 5. Primer sequences for qRTPCR

Gene	Probe number	Primer sequece	
Drd1	82	Fwd	tctggtttacctgatccctca
		Rev	gcctcctccctcttcaggt
Drd2	71	Fwd	gatgcttgccattgttcttg
		Rev	attcaggatgtgcgtgatga
Drd3	9	Fwd	ctagtggtgagcctggctgt
		Rev	ccagactccacctgtcacct
Drd4	92	Fwd	cctctctttgtctactccgaggt
		Rev	gccatgagcgtgtcacag
Drd5	18	Fwd	tcctggtgtgcttatgctttc
		Rev	tcagctaagaatcgtttggtttc
Nppa	25	Fwd	cacagatctgatggatttcaaga
		Rev	cctcatcttctaccggcatc
Nppb	45	Fwd	cccctactccgtgaaaaggt
		Rev	tcttcaacaacggtgcctct
HPRT	22	Fwd	atcagtcaacgggggacata
		Rev	caacaatcaagacattctttcca

Supplementary Table 5: Primer sequences for qPCR

Primer sequences for qPCR. Probe number, Universal Probe library probe number; Fwd, forward sequencing primer; Rev, reverse sequencing primer
