# Supplementary material for: Cardiac dopamine D1 receptor triggers ventricular arrhythmia in chronic heart failure
Source: Nat Commun. 2020 Aug 31;11:4364. doi: 10.1038/s41467-020-18128-x (PMC7459304; doi:10.1038/s41467-020-18128-x)
Supplement: Supplementary file 3 — Reporting Summary [file 41467_2020_18128_MOESM3_ESM.pdf]

nature research

Corresponding author(s): Atsuhiko T.Naito

Last updated by author(s): Jun 9, 2020

Reporting Summary

Nature Research wishes to improve the reproducibility of the work that we publish. This form provides structure for consistency and transparency in reporting. For further information on Nature Research policies, see our Editorial Policies and the Editorial Policy Checklist.

Statistics

For all statistical analyses, confirm that the following items are present in the figure legend, table legend, main text, or Methods section.

n/a	Confirmed	
[ ]	[x]	The exact sample size (*n*) for each experimental group/condition, given as a discrete number and unit of measurement
[ ]	[x]	A statement on whether measurements were taken from distinct samples or whether the same sample was measured repeatedly
[ ]	[x]	The statistical test(s) used AND whether they are one- or two-sided<br>*Only common tests should be described solely by name; describe more complex techniques in the Methods section.*
[x]	[ ]	A description of all covariates tested
[x]	[ ]	A description of any assumptions or corrections, such as tests of normality and adjustment for multiple comparisons
[ ]	[x]	A full description of the statistical parameters including central tendency (e.g. means) or other basic estimates (e.g. regression coefficient) AND variation (e.g. standard deviation) or associated estimates of uncertainty (e.g. confidence intervals)
[ ]	[x]	For null hypothesis testing, the test statistic (e.g. *F*, *t*, *r*) with confidence intervals, effect sizes, degrees of freedom and *P* value noted<br>*Give P values as exact values whenever suitable.*
[x]	[ ]	For Bayesian analysis, information on the choice of priors and Markov chain Monte Carlo settings
[x]	[ ]	For hierarchical and complex designs, identification of the appropriate level for tests and full reporting of outcomes
[ ]	[x]	Estimates of effect sizes (e.g. Cohen's *d*, Pearson's *r*), indicating how they were calculated

Our web collection on statistics for biologists contains articles on many of the points above.

Software and code

Policy information about availability of computer code

Data collection	Zeiss ZEN 2009(Zeiss), Vevo 2100 imaging system(Visualsonics Inc.), Data Insights(PONEMAH v5.20, DSI), In Cell Developer Toolbox 1.9.1 (GE Healthcare)
Data analysis	GraphPad Prism 6.07

For manuscripts utilizing custom algorithms or software that are central to the research but not yet described in published literature, software must be made available to editors and reviewers. We strongly encourage code deposition in a community repository (e.g. GitHub). See the Nature Research guidelines for submitting code & software for further information.

Data

Policy information about availability of data

All manuscripts must include a data availability statement. This statement should provide the following information, where applicable:

- Accession codes, unique identifiers, or web links for publicly available datasets
- A list of figures that have associated raw data
- A description of any restrictions on data availability

Publicly available datasets(GSE116250,46224,95143, and 29446) were analyzed. Other original data showing in this study is available in the Source data file. Further data will be available upon request.

Field-specific reporting

Please select the one below that is the best fit for your research. If you are not sure, read the appropriate sections before making your selection.

- [x] Life sciences
- [ ] Behavioural & social sciences
- [ ] Ecological, evolutionary & environmental sciences

For a reference copy of the document with all sections, see nature.com/documents/nr-reporting-summary-flat.pdf

Life sciences study design

All studies must disclose on these points even when the disclosure is negative.

Sample size	No statistical methods were used to predetermine sample size estimates. Sample size was determined based on the experimental results that we obtained from preliminary experiments and published papers. In vivo studies, we used standard sample sizes reported in the literature previously in mouse studies. The numbers of performed experiments were indicated in each figure legend.
Data exclusions	Before starting in vivo experiments, we have established the criteria that mice died within 1 week after the operation were excluded from the analysis.
Replication	Major experiments were performed with an appropriate sample size to obtain statistical significance. To keep the number of animals used in experiments as low as possible, animal experiments related with arrhythmia was performed with limited sample size. However, we performed two different method for analyzing arrhythmia to obtain reproducible phenotype. To make sure that the experiments could be reproduced, more than one person was often involved in major experiments (e.g. RNA-seq analysis or smFISH analysis) and human data was confirmed by two independent hospital with international collaboration.
Randomization	In vivo experiments, mice were randomly allocated to each group. There was no randomization for in vitro experiments.
Blinding	Clinical histories of human subjects were obtained by independent operators who were blinded to Drd1 expression level. There was no blinding for in vitro cellular experiment.

Reporting for specific materials, systems and methods

We require information from authors about some types of materials, experimental systems and methods used in many studies. Here, indicate whether each material, system or method listed is relevant to your study. If you are not sure if a list item applies to your research, read the appropriate section before selecting a response.

Materials & experimental systems

n/a	Involved in the study	
☐	☒	Antibodies
☒	☐	Eukaryotic cell lines
☒	☐	Palaeontology and archaeology
☐	☒	Animals and other organisms
☐	☒	Human research participants
☐	☒	Clinical data
☒	☐	Dual use research of concern

Methods

n/a	Involved in the study	
☒	☐	ChIP-seq
☒	☐	Flow cytometry
☒	☐	MRI-based neuroimaging

Antibodies

Antibodies used	anti-DDDDK antibody PM020; Medical & Biological Laboratories Co., Ltd., Nagoya, Japan<br>anti-phospso-RyR2(Ser2808 and Ser2814) antibody; Badrilla, Ltd. United Kingdom<br>anti- β-actin antibody ; #MA5-11869, Thermo Fisher Scientific Inc. Waltham, MA USA<br>The dilution of antibodies used in the study is described in Method section.
Validation	anti-DDDDK antibody is validated for Immunocytochemistry, WB, IP by the manufacturer and many investigator in the literature. (Iimori, M., et al., Nat. Commun. 7, 11117(2016), Takahashi S., et al. J Cell Sci. 122, 985-94 (2009), Murata H., et al., J Biol Chem. 293, 18933-18943 (2018))<br>anti-phospso-RyR2(Ser2808 and Ser2814) antibody is validated for WB by other investigator in the literature.(Okuda, S., et al., BBRC 496, 1250-1256 (2018), Sadredini M., et al., PLoS ONE. 2016;11:e0153887, Kim E., et al., J Clin Invest. 2014;124:5027-36, Besser J., et al., PLoS ONE. 2014;9:e113449)<br>anti-β-actin antibody is validated for IHC, IP, WB, ICC, ELISA by the manufacurer and many other investigators. (Baoyu Zhao, et al., Nature. 2019 May; 569(7758): 718–722. Nathan R., et al., Nat Commun. 2019; 10: 3790., Nima Borhan F., et al., Sci Rep. 2017 Oct 2;7 (1):12480.)

Animals and other organisms

Policy information about studies involving animals; ARRIVE guidelines recommended for reporting animal research

Laboratory animals	Mice were housed in a specific pathogen-free facility with a 12-h light/12-h dark cycle. Ambient room temperature was regulated at 73±5 °F and humidity was controlled at 50±10%. C57BL/6 were purchased from CLEA JAPAN. C57BL/6-Drd1atm1a(KOMP)Wtsi mice were purchased from the KOMP Repository and crossed with C57BL/6-Tg(CAG-Flpe)2 Arte to delete the neomycin resistance gene and obtain Drd1flox/+ heterozygous mice.<br>Myh6-Cre mice were purchased from Jackson Laboratory (stock#009074, stock name: STOCK Tg (Myh6-cre)1Jmk/J) . B6.Cg-Drd1atm1(tTA)Mok, B6.Cg-Tg(TRE-D1d1a/lacZ)1Mok and B6.Cg-Myh6(tTA)Mok mice were generated from our laboratories(Chiken, S., et al. 2015) (Sanbe, A., et al. 2009). Age of mice operated and used for experiments were 8 to 12-week old male mice.
Wild animals	The study did not involve wild animals.
Field-collected samples	The study did not involve field collected samples.
Ethics oversight	The University of Tokyo Ethics Committee for Animal Experiments.

Note that full information on the approval of the study protocol must also be provided in the manuscript.

Human research participants

Policy information about studies involving human research participants

Population characteristics	In University of Tokyo, heart tissues were obtained immediately after death due to non-cardiac cause (one patient with normal cardiac function) or during left ventricular assist device surgery or heart transplantation (11 patients with cardiomyopathy). We presented the population characteristics of the human research participants in Supplementary table 2 as per editors' recommendation.<br>In the University of Colorado, explanted failing hearts were collected from adult patients undergoing heart cardiac transplantation at the University of Colorado Hospital as part of the Division of Cardiology Cardiac Tissue Biobank under a long-standing protocol approved by the Colorado Multiple Institutional Review Board (COMIRB, protocol 01-568) where transplant-listed patients signed written consent for use of their explanted hearts for research purposes. Clinical characteristics of the cohorts were written in the previously described paper (Sweet et al., BMC Genomics. (2018) 19:812)
Recruitment	The recruitments and the collection of clinical histories were performed by independent operators who were blinded to the results of RNA-seq analysis about DRD1.
Ethics oversight	All experiments were approved by the ethics committee of the University of Tokyo (G-10032) and the Colorado Multiple Institutional Review Board (COMIRB, protocol 01-568) respectively. All patients gave written informed consent before taking part in the study.

Note that full information on the approval of the study protocol must also be provided in the manuscript.

Clinical data

Policy information about clinical studies

All manuscripts should comply with the ICMJEguidelines for publication of clinical research and a completedCONSORT checklist must be included with all submissions.

Clinical trial registration	*Provide the trial registration number from ClinicalTrials.gov or an equivalent agency.*
Study protocol	*Note where the full trial protocol can be accessed OR if not available, explain why.*
Data collection	*Describe the settings and locales of data collection, noting the time periods of recruitment and data collection.*
Outcomes	*Describe how you pre-defined primary and secondary outcome measures and how you assessed these measures.*
